# Plural Pregnancy, Involving a Question as to Sire

**Published:** 1895-11

**Authors:** W. H. Harbaugh

**Affiliations:** Richmond, Va.


### PLURAL PREGNANCY, INVOLVING A QUESTION AS TO SIRE.

BY DR. W. H. HARBAUGH,
RICHMOND, VA.

(Concluded from page 638 )

Last winter I was called to treat the well-known mare, Miss Lasitter, at Whitby Stock Farm. The day before she had slipped two colts that were apparently the result of two conceptions with a considerable interval between them, one being about twice the size of the other, but the smaller having no appearance of being a withered fœtus. This mare was served by Norfolk on March 9, 1894, after which she was tried to him, and refused him six different times, and then she came in heat again and took him on May 21st. If impregnation followed each one of these copulations, and there is no impossibility connected with the idea, there was a difference of about seventy-three days between the two conceptions. The foals were slipped on January 26, 1895. Three hundred and twenty-three days had elapsed since the first service, and two hundred and fifty days since the second. Multiple pregnancies usually terminate before the full term, which is supposed to be caused by the overdistention of the uterus, which excites contractions at an earlier period than in single pregnancies.

In the mare 300 to 400 days are considered to be the extreme limits of normal gestation, while the average duration of gestation is from 340 to 350 days.

Lord Spencer recorded the following observations: Of 102 mares, 3 foaled on the 311th day; 1 foaled on the 314th day; 1 foaled on the 325th day; 1 foaled on the 326th day; 2 foaled on the 330th day; 47 foaled between the 340th day and the 350th day; 25 foaled between the 356th day and the 360th day; 21 foaled between the 360th day and the 377th day; 1 foaled on the 394th day. Between the extremes there is a period of 83 days, and even if the earlier ones are called premature births there still remains much difference with the rest.

In cases of twins where one is born prematurely and the other carried to the full term the immaturity of the first may be overlooked, but in the case of the mare Miss Lasitter, both being born at the same time, the immaturity of one was the remarkable point, while the other was sufficiently matured to be reared if he had been born alive. In instances of twins born alive it must be remembered that very often one is better developed than the other, one may be strong and the other weak and puny, and such instances are always cited against superfecundation, but it must also be admitted that the same is the case with single births, some children by the same parents being born fat and large while others are small and weak. Physiologists say that it is exceptional that mature young are born earlier than usual, and that when the intra-uterine development is slow the fœtus is carried longer than the average time. This theory may be used in support of different arguments.

I am of opinion that the case of the mare Miss Lasitter was another instance of superfecundation, and that the time between the two conceptions—73 days—was the only remarkable thing about it. In the case of the brown mare, however, a question as to sire is involved, which makes the case doubly interesting. I am of opinion she was impregnated by Norfolk on March 1st or April 1st, and by Eggwood on April 30th, thus making an interval of one or two months between the conceptions. That each colt resembled his sire must not be considered a mere coincident, as it is generally conceded that colts resemble their sire in color as well as conformation, and that fillies resemble their dams.

There is no physiological or anatomical impossibility in a second impregnation up to the time that the decidua reflexa and the decidua vera become intimately united in the human female, and there is no such impossibility in the mare up to the time that the embryo or fœtus so completely fills the uterus as to obstruct the os uteri as well as the uterine openings of the Fallopian tubes.

In the uterus of a woman opened about the fifth week of pregnancy the tumor is found in or near the fundus, projecting into the cavity of the uterus. The Fallopian tubes are open. The membrane covering the tumor is found to be identical in structure and continuous with the mucous membrane of the uterus. In the tumor is a cavity containing an impregnated ovum. This leads us to the formation of the decidual membrane. The impregnated ovum finds its way into the uterus from the Fallopian tube, where it remains free for a while, and is then probably arrested in the convolutions of the uterine mucous membrane, which gradually rise round it and unite, forming the decidua reflexa, the mucous membrane of the uterine cavity being the decidua vera. Hence, during the stage it is not an anatomical impossibility for another ovum to be impregnated and develop along with the first. As the embryo enlarges and fills the uterus the decidua reflexa and the decidua vera become united and difficult to separate. I will better explain the formation and development of the decidua on the blackboard.

As I said before, superfœtation is defined differently by different persons, and Scanzoni, for instance, says that it must mean that superfecundation has occurred after the decidua vera and decidua reflexa have come in contact. If this definition is accepted, I doubt very much if superfœtation ever occurs, as the uterine cavity is completely filled when the decidua becomes united, and consequently there is little chance of the spermatozoa reaching an ovum in the Fallopian tube.

In the mare the decidua is not formed, but as the embryo enlarges the chorion becomes attached to the uterine membrane gradually, till the uterine cavity is filled, and also the horns of the uterus to a great extent, one, of course, being filled to a greater extent than the other.

I have here an embryo (preserved in alcohol) which was passed by a mare exactly four weeks after she had been served by the stallion. You will observe that it is very small, about one inch in length; you will also conclude that this small thing, with its envelopes, would not form a very large tumor in the uterus of a mare; certainly not large enough to fill the uterus sufficiently to prevent the spermatozoa reaching the Fallopian tubes. But small as it is, its outlines are well marked, as we can plainly discern legs, tail, ribs, tongue, and nostrils. We are told that at the second period of development, toward the twenty-eighth day, the embryo of the mare measures about six lines, but here we have one about twice that length, and it is certainly not more than a twenty-eighth-day embryo, and may be less, as the ovum may not have been fecundated for a day or two after copulation. From the fifth to the eighth week is called the third period of development, during which time the embryo of the mare is said to measure a little more than two inches, which is still not large enough to fill the uterus completely. The fourth period is given as from the ninth to the thirteenth week, and the measurement is said to be about six inches. From the fourteenth to the twenty-second week is called the fifth period, during which time the fœtus of the mare is said to measure about thirteen inches.

During any of the periods up to the fifth I cannot see any anatomical reason why superfecundation cannot occur. The sixth period is from the twenty-third to the thirty-fourth week, during which time the fœtus of the mare reaches a length of over two feet. The seventh or last period is from the thirty-fifth to the forty-eighth week, or till the end of gestation. The average length of a foal at birth is about three and a half feet.

In human anatomy the impregnated ovum is called an embryo for the first three months, after which time it is called a fœtus. I can see no objection to using the term superfecundation in those cases where a second fecundation has occured within the first three months, and the term superfœtation in the cases where it is supposed that a second conception has occurred more than three months after the first.

It is certain that the orifices of the Fallopian tubes remain open during the early months of pregnancy; it is certain that mares occasionally go in heat during pregnancy; and it is also certain that the function of the ovaries is not always stopped by pregnancy, and, therefore, there remains nothing but the mucous plug in the cervix to prevent a second conception, and this, it must be admitted, is not a sufficient bar to the entrance of spermatozoa, especially when the organs of generation are excited by the condition called heat. Consequently, cases of superfecundation are not to be looked upon as wonderful; the real wonder lies in the direction that more instances are not reported.

The doctrine of superfœtation would be supported by cases where one mature child is born, followed by the birth of another mature child months after the birth of the first.

A woman at Palermo was delivered of a male child on November 12th, and on February 2d was delivered of another male child, both perfect. A woman was delivered of a girl on October 16th, well formed and apparently mature, and on November 18th another was delivered, healthy and "showing no signs of overmaturity." I must remark that the reporter of this case does not inform us what signs we should look for in order to discover what he terms overmaturity. Dr. Bounar (reported by Leishman) "gives three cases, all occurring in families of rank and position. In these cases the intervals between the two deliveries were 182, 174, and 127 days, and all the children were sufficiently developed to be reared, and without exception to reach maturity. . . . . We know that impregnation may occur within a very short period of delivery. . . . . But in the case last mentioned this would only give about four calendar months from an impregnation assumed to date six days after the last delivery, an age at which it would not be possible to rear the child." Superfœtation may occur in cases of extra-uterine pregnancy, as well as in females with a double uterus.

A very interesting case (reported by Leishman) occurred in the practice of Dr. J. Harris Ross, of Scotland. On July 16th he delivered a woman of twins of between five and six months growth. At this delivery he discovered a second opening to the left of the os from which the twins were delivered, which, at the time, he supposed to be an excavated ulcer. On October 31st a child, full grown, was delivered from this left opening, which proved to be the os of a corresponding uterine cavity. Examination disclosed a complete septum between the two cavities of the uterus. The woman had menstruated three times since the birth of the twins in July. Mother and child recovered. This case shows that an ovum from each ovary was fecundated, and that while a child was developing in one cavity the other cavity was performing its physiological function of menstruation.

While some of the foregoing cases are really only cases of superfecundation, some of them may be cases of superfœtation, but as yet superfœtation may be considered but a theory to explain some rare cases met with in obstetrical practice. On the other hand, superfecundation may be a fact in any seeming ordinary case of plural pregnancy.

